# Human Mesenchymal Stromal Cells Resolve Lipid Load in High Fat Diet-Induced Non-Alcoholic Steatohepatitis in Mice by Mitochondria Donation

**DOI:** 10.3390/cells11111829

**Published:** 2022-06-02

**Authors:** Sandra Nickel, Madlen Christ, Sandra Schmidt, Joanna Kosacka, Hagen Kühne, Martin Roderfeld, Thomas Longerich, Lysann Tietze, Ina Bosse, Mei-Ju Hsu, Peggy Stock, Elke Roeb, Bruno Christ

**Affiliations:** 1 Department of Visceral, Transplant, Thoracic and Vascular Surgery, University of Leipzig Medical Center, 04103 Leipzig, Germany; sandra.nickel@medizin.uni-leipzig.de (S.N.); madlen.christ@medizin.uni-leipzig.de (M.C.); sandra.winkler.halle@gmail.com (S.S.); joanna.kosacka@medizin.uni-leipzig.de (J.K.); hagen.kuehne@freenet.de (H.K.); lysann.tietze@medizin.uni-leipzig.de (L.T.); ina.bosse@medizin.uni-leipzig.de (I.B.); hsumeiju@gmail.com (M.-J.H.); peggy.stock@medizin.uni-leipzig.de (P.S.); 2 Division of General, Visceral and Vascular Surgery, University Hospital Jena, 07747 Jena, Germany; 3 Department of Gastroenterology, Justus-Liebig-University, 35392 Giessen, Germany; martin.roderfeld@innere.med.uni-giessen.de (M.R.); elke.roeb@innere.med.uni-giessen.de (E.R.); 4 Institute of Pathology, Heidelberg University Hospital, 69120 Heidelberg, Germany; thomas.longerich@med.uni-heidelberg.de

**Keywords:** mesenchymal stromal cells, non-alcoholic steatohepatitis, cell transplantation, mitochondria transfer

## Abstract

Mesenchymal stromal cells (MSC) increasingly emerge as an option to ameliorate non-alcoholic steatohepatitis (NASH), a serious disease, which untreated may progress to liver cirrhosis and cancer. Before clinical translation, the mode of action of MSC needs to be established. Here, we established NASH in an immune-deficient mouse model by feeding a high fat diet. Human bone-marrow-derived MSC were delivered to the liver via intrasplenic transplantation. As verified by biochemical and image analyses, human mesenchymal stromal cells improved high-fat-diet-induced NASH in the mouse liver by decreasing hepatic lipid content and inflammation, as well as by restoring tissue homeostasis. MSC-mediated changes in gene expression indicated the switch from lipid storage to lipid utilization. It was obvious that host mouse hepatocytes harbored human mitochondria. Thus, it is feasible that resolution of NASH in mouse livers involved the donation of human mitochondria to the mouse hepatocytes. Therefore, human MSC might provide oxidative capacity for lipid breakdown followed by restoration of metabolic and tissue homeostasis.

## 1. Introduction

The incidence of non-alcoholic fatty liver diseases (NAFLD) is about 25% worldwide, which thus constitutes a major health problem [1]. Principally, hepatic lipid storage is reversible. However, depending on multiple pathomechanisms, NAFLD may convert to an inflammatory phenotype (non-alcoholic steatohepatitis, NASH) eventually promoting liver fibrosis, cirrhosis and hepatocellular carcinoma (HCC) [2,3]. Today, NASH increasingly contributes to the incidence of liver cancer worldwide, being the second most frequent cause of cancer-related death. The pathogenesis of NASH is multi-faceted, comprising besides others, nutritional, behavioral and genetic predispositions [4,5,6]. Histologically, fat-laden hepatocytes, inflammatory infiltrates, ballooning and death of hepatocytes are prevailing signs of NASH in the liver, while collagen fiber deposition indicates disease progression [7,8,9]. On the molecular level, the metabolic syndrome in association with insulin resistance seems a key player in NASH, driving activation of sterol regulatory element-binding protein 1 (SREBP-1c), the major activator of hepatic de novo lipogenesis and triglyceride synthesis. In addition, augmented lipolysis in the adipose tissue floods the liver with free fatty acids, fueling into hepatic triglyceride stores. The resulting lipid overload has consequences for hepatocyte fatty acid metabolism, rendering mitochondria, microsomal and peroxisomal fatty acid oxidation at its metabolic limit. This causes oxidative stress associated with lipid peroxidation, activation of inflammatory pathways in hepatocytes and non-parenchymal cells and eventually hepatocyte death (for recent reviews cf. [10,11,12]).

Mesenchymal stromal cells (MSC) carry big options of treatment of inflammatory diseases due to their anti-inflammatory and immune-modulatory potential [13,14,15]. In rodents, they ameliorated hepatic damage and supported tissue regeneration in models of acute and chronic liver diseases such as acute liver failure due to paracetamol intoxication [16,17] and liver cirrhosis induced by chronic carbon tetrachloride exposure [18,19]. In mouse models of diet-induced NASH, MSC improved tissue homeostasis by lowering hepatic lipid storage and fibrosis. In mice, the artificial methionine-choline-deficient (MCD) diet rapidly induces the steatotic, fibrotic and inflammatory liver phenotype. However, systemic signs of NASH such as, e.g., the metabolic syndrome in association with insulin resistance, are missing [20,21]. Feeding of a high fat diet over weeks to months induces a NASH phenotype associated with systemic features such as obesity and insulin resistance. In these models, MSC improved both the liver and the systemic phenotype by reducing hepatic fat accumulation, normalizing serum triglycerides and cholesterol and re-establishing insulin sensitivity [22,23,24]. Mechanistically, however, the mode of MSC action remains largely elusive. Recent reports identified lipotoxicity and ER stress as MSC targets, which were inhibited by MSC treatment of rats suffering from high-fat-diet-induced NASH [25]. In a mouse model of NASH-associated diabetes, an MSC-conditioned medium improved both NASH and insulin sensitivity by targeting mitochondria [26]. This is interesting, since mitochondrial dysfunction and mitochondria biogenesis contribute to the pathogenesis of NASH [27,28,29]. In line, in the mouse model of MCD-diet-induced NASH, the donation of mitochondria from human MSC transplants to mouse hepatocytes was suggested to provide oxidative capacity to host hepatocytes, thereby restoring mitochondrial fatty acid oxidation from triglycerides, thus decreasing hepatocyte lipid storage [30]. It is unknown whether mitochondria donation from MSC to mouse hepatocytes may represent a primary action of MSC to reduce hepatic lipid load followed by secondary improvement of tissue and metabolic homeostasis by, e.g., amelioration of lipid-induced oxidative stress, inflammation and fibrosis besides others. If this is true, the improvement of NASH by MSC transplantation should be independent of the pathomechanisms leading to NASH.

To verify this hypothesis, in the present study we applied the more physiologic mouse model of high-fat-diet-induced NASH. We show that human MSC ameliorate NASH-associated lipid load and that mouse hepatocytes harbor human mitochondria. We conclude that human MSC donate mitochondria to mouse hepatocytes, thereby providing oxidative capacity for lipid breakdown. Since we also observed this phenomenon in a mouse model of MCD-diet-induced NASH [30], it is thus independent of the etiology of NASH. The pleiotropic actions of MSC to improve NASH are likely not mediated by the same pleiotropic mechanisms. Therefore, mitochondria donation may represent a superordinate primary level of MSC action, which secondarily mediates the versatile mechanisms involved in the amelioration of NASH.

## 2. Materials and Methods

### 2.1. Isolation of MSC from Human Bone Marrow

Methods to isolate and process human bone-marrow-derived MSC as well as their hepatocytic differentiation have been described in detail previously [31]. These procedures yielded reproducible batches of cells expressing a set of mesenchymal markers and featuring multiple differentiation capacity as described in detail in our previous studies [32,33]. Cells were harvested from bone marrow waste material from elective knee or hip surgeries after obtaining written consent by the donors. Procedures were approved by the Institutional Ethics Review Board Leipzig (file no. 282/11-lk). The use of human liver slices was approved by the Institutional Ethics Review Board Leipzig (file no. 331-15-24082015 in its latest version 331/15-ek dated 24 January 2019).

### 2.2. Animals and MSC Application

For transplantation of human bone-marrow-derived mesenchymal stromal cells, immunodeficient Pfp/Rag2^−/−^ mice (C57BL6, B6.129S6-Rag2(tm1Fwa)Prf1(tm1Clrk)N12. Taconic; Ejby, Denmark) were used. The mice were 12–16-week-old males that were fed either a high fat diet (HFD; No. S7803-E750, ssniff Spezialdiäten GmbH, Soest, Germany), or a standard rodent diet (ND; No. V1324, ssniff Spezialdiäten GmbH, Soest, Germany). A total of 21 weeks after the start of feeding, 1/3 partial hepatectomy was performed to stimulate hepatocyte proliferation to foster cell transplant enrichment in the host liver. Where indicated, mice received 0.9–1 × 10^6^ human MSC (hepatocytic differentiated) via splenic injection as described before [21]. PBS was injected as the solute control. Finally, the four experimental groups represented ND − MSC, ND + MSC, HFD − MSC and HFD + MSC. After surgery, feeding of the high fat diet or the control diet was continued for 7 days. Then, the mice were sacrificed and livers explanted for downstream analyses. Housing conditions were: 12 h light/dark cycle, ambient temperature of 22 °C ± 2 °C, humidity 50–60%. All experimental procedures involving animals were approved by the federal authorities of Saxony (file no. TVV_54_16) and followed the guidelines of the animal welfare act.

### 2.3. Immunohistochemical Procedures

Antibodies used in this study, their distributors and dilutions are listed in Table 1. Tissue slices of 1 µm were generated from livers after overnight fixation in 4% paraformaldehyde and embedding in paraffin. For immunohistochemical detection of HepPar1, epitope retrieval was performed under heating in a citrate buffer (pH 6.0) for 30 min followed by peroxidase blocking in 3% hydrogen peroxide/methanol for 20 min and by a subsequent 60 min blocking step in 5% BSA/0.5% Tween20. Slices were incubated overnight with the HepPar1 antibody at 4 °C. The secondary HRP goat anti-mouse Ig was offered for 90 min at room temperature followed by DAB chromogen (Thermo Fisher Scientific, Dreieich, Germany) incubation and counterstaining with nuclear fast red-aluminum sulfate solution (0.1%). Finally, slices were embedded in Entellan (Merck GmbH, Darmstadt, Germany).

For immunofluorescent detections, antigens were demasked in Tris-EDTA buffer (pH 9.0) for 30 min followed by blocking in 5% goat serum/PBS (ccpro, Oberdorla, Germany) for 20 min and in 5% BSA/0.5% Tween20 for 60 min. Primary antibodies to detect perilipin 1, E-cadherin, β-catenin, N-cadherin, ZO-1, THBS1 and CD42b were offered overnight at 4 °C. THBS1 and CD42b were then detected with secondary antibodies goat anti-mouse Cy3 and goat anti-rabbit AlexaFluor 488 for 1 h at room temperature. Co-detection of N-cadherin and ZO-1 was achieved by combining goat anti-mouse Cy3 and goat anti-rabbit AlexaFluor 488. For co-detection of E-cadherin and β-catenin, goat anti-mouse AlexaFluor 488 and goat anti-mouse Cy3 were used in combination with the M.O.M kit (Vector, BIOZOL GmbH, Eching, Germany). Nuclei were counterstained with DAPI (1:1000, Carl Roth GmbH + Co. KG, Karlsruhe, Germany) for 5 min and slices mounted in 50% glycerol solution and lacquer. The Zeiss Axio Observer.Z1 microscope was used for imaging.

Human mitochondria were detected in mouse hepatocytes using an anti-human mitochondria antibody detecting an outer membrane protein on intact human mitochondria. Slices were heated for 30 min in a citrate buffer (14746, SignalStain unmasking solution, Cell Signaling Technology, Frankfurt/Main, Germany) followed by cooling on ice for 30 min. Slices were then washed twice in PBS and blocked for 80 min in 5% goat serum and 0.3% TritonX-100 in PBS. Primary antibodies against mouse cyclophilin A and anti-human mitochondria were offered overnight at 4 °C followed by 3 washes with PBS. Then, the goat anti-mouse Cy3 antibody was added for 60 min at room temperature followed by two washes in PBS for 10 minutes each. The goat anti-rabbit AlexaFluor 488 antibody was added afterwards for 60 min at room temperature. After 3 washings with PBS each for 5 min, nuclei were stained with DAPI. Finally, slices were mounted with 50% glycerol solution and lacquer. Images were captured with a Zeiss Axio Observer.Z1 microscope equipped with ApoTome.2 at the 40× magnification.

The number of mitochondria was quantified using the ImageJ software (ImageJ 1.48, National Institutes of Health, Bethesda, MD, USA). From each animal, 5–8 fields on a microscopic picture (40× magnification) were analyzed. To correct for background signals, a threshold range was set from 72 to 255. Using the scale bar (100 µm), the size of a visual field was calculated as 0.0376 mm^2^, and pixel length was 6.2 pixel/µm. Assuming a maximum mitochondrion length of 4.5 µm, only particles up to a size of 20 pixel^^2^ were counted, while particles greater than 20 pixel^^2^ were excluded.

### 2.4. Histopathological Scoring

Histological scoring of NAFLD of formalin-fixed paraffin-embedded murine liver tissues was performed using the non-alcoholic liver disease Activity Score (NAS) [34] and the Steatosis-Activity-Fibrosis (SAF) [35], respectively. Applying the NAS, the disease typing was performed as follows: NASH (NAS ≥ 5), borderline or possible NASH (NAS = 3 or 4), no NAFLD (NAS < 3 or steatosis < 5%). Using the SAF score, NAFLD was defined by the presence of steatosis in >5% of hepatocytes and NASH by the additional presence of both hepatocellular ballooning and lobular inflammation of any degree.

### 2.5. Semiquantitative PCR

The mRNA expression of mouse marker genes of lipid utilization (FABP1, PPARα, ACOX1), β-oxidation (ACSL1, ACAA2, CPT1) and triglyceride synthesis (FASN, MOGAT1) was measured by semi-quantitative RT-PCR. Total RNA was isolated using a standard Trizol protocol and 4µg of RNA for reverse transcription with the Maxima-H-Minus-First-Strand kit (Thermo Fisher, Dreieich, Germany). PCR was carried out using the PCR Master Mix (2×) (Thermo Fischer Scientific, Dreieich, Germany) and mouse (m) and human (h) primers as listed in Table 2. After electrophoretic separation, the PCR products were analyzed by quantifying the relative intensity of specific bands using the Gel Analyzer software (Media Cyberneties, Silver Spring, MD, USA); β2-microglobulin expression was used for normalization of loading differences.

### 2.6. Quantification of Liver Triglyceride Content

Frozen liver tissue (100 mg) was minced with a pestle in 1 mL of 5% Igepal (I3021, Sigma-Aldrich, Taufkirchen, Germany) and heated for 4 min at 95 °C using a thermomixer. After cooling at ambient temperature, samples were heated again and then spun down at maximum speed in a microcentrifuge. Supernatants were diluted in 10 volumes of distilled water; 25 µL of the pre-warmed (37 °C; 1–5 min) samples were analyzed using the Triglyceride Assay Kit-Quantification (ab65336, Abcam, Berlin, Germany) as described in the supplier manual.

For image analysis of lipids, cryosections (8–10 µm) were stained with Sudan III (4711.1, Carl Roth GmbH, Karlsruhe, Germany) for 20 min, washed two times in phosphate-buffered saline and counterstained with hemalum. Images were captured with a Zeiss Axio Observer.Z1 inverted microscope at the 20× magnification. The amount of lipids was estimated as the percentage amount of stain per visual field out of 6–10 microscopic fields per group by image analysis using the ImageJ software (ImageJ 1.42, National Institutes of Health, USA).

### 2.7. Multiplex Assay

Mouse Magnetic Luminex^®^ Assay (LXSAMSM, R&D Systems, Minneapolis, MN, USA) was performed to quantify hepatic protein levels of MMP-12 and TIMP-1. Briefly, 20 mg liver tissue was homogenized in 400 µL PBS buffer containing 1% Triton X-100 (Sigma-Aldrich Chemie GmbH, Taufkirchen, Germany) and a protease inhibitor cocktail (cOmplete Mini, Hoffmann-La Roche, Basel, Switzerland) using a T8 ULTRA-TURRAX (IKA-Werke, Staufen, Germany) on blue ice. After centrifugation, 200 μL of the supernatant was diluted in 400 μL of the kit’s calibrator diluent RD6-52. Measurements were performed according to the manufacturer’s instructions using a Luminex^®^ 200 flow-cytometer and xPONENT^®^ software, version 3.1 (Luminex Corp., Austin, TX, USA).

### 2.8. Statistics

SPSS23 or SigmaStat were used for statistical analysis. Data were tested for normal distribution using the Kolmogorov–Smirnov and Shapiro–Wilk tests. Data transformation to normal distribution was performed using the Johnson transformation. For the quantification of lipids with Sudan III, data are normally distributed, and the two-sided Student’s *t*-test was applied. Significant differences between the groups in the mRNA expression analysis were validated by one-way ANOVA and the Newmann–Keuls test. Data of collagen quantification are not normally distributed; the significance analysis was performed using the Kruskal–Wallis algorithm. The Bonferroni test was used as a post hoc analysis. The data set concerning E-cadherin expression is normally distributed, and the One-way ANOVA and LSD post hoc tests were performed. The data set shown for the expression of TIMP-1 and MMP-12 was brought into normal distribution by means of the Johnson transformation. Then a one-way ANOVA and a LSD post hoc test were used to analyse group differences. The data set in Figure S3 is normally distributed, and significance analysis was performed by one-way ANOVA with post hoc LSD. Statistics for mitochondria amount were calculated using the Kruskal–Wallis algorithm and post hoc Bonferroni. More specific details to calculate statistical significance are described in the legends to figures where appropriate.

## 3. Results

### 3.1. Hepatic Transplantation of MSC Decreases NASH-Associated Hepatic Lipid Storage

**Figure 1:**
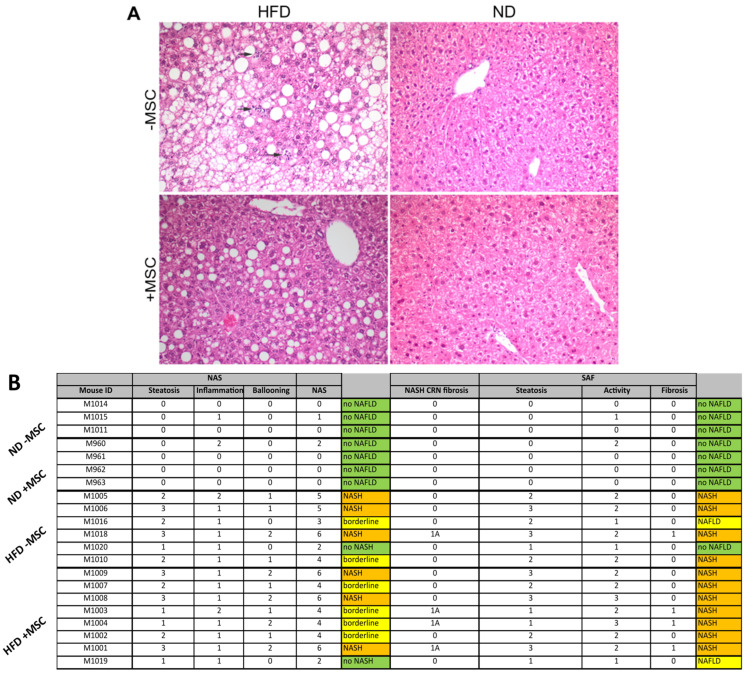
High-fat-diet-fed mice develop NASH: (**A**) HE stain of liver slices from animals fed either the control (ND) or the high fat diet (HFD); where indicated, animals received MSC (+MSC) or were left untreated (−MSC). Arrows indicate inflammatory foci. Pictures are representative for 2 different animals out of each group. (**B**) Determination of NASH scores using either the NAS [34] or the SAF [35] score; 3 (ND − MSC), 4 (ND + MSC), 6 (HFD − MSC) and 8 (HFD + MSC) animals per group are included for analysis. Numbers in columns represent the score according to the respective scoring system used (cf. Materials and Methods). The pathological evaluation of the scores revealed either no NAFLD (green), NASH (orange), or borderline and NAFLD (yellow).

The high fat diet increased hepatic lipid accumulation compared to mice fed the control diet. MSC treatment reduces the lipid content compared to mice receiving the high fat diet only (Figure 1A). To evaluate whether the high fat diet also induces NASH in our model and whether MSC treatment acts as a preventive, we scored the murine fatty liver disease using two well established histopathological scoring systems, the **N**AFLD **A**ctivity **S**core (NAS, [34]) and the **S**teatosis-**A**ctivity-**F**ibrosis score (SAF, [35]). A high fat diet induces a robust NASH phenotype, including steatosis, hepatocyte ballooning and inflammation. In addition, MSC treatment seems to ameliorate NASH leading to a borderline disease between NAFLD and NASH as suggested by the NAS. However, when the SAF score was applied, the NASH phenotype was not rescued by MSC treatment (Figure 1B), though lipid accumulation was obviously attenuated.

**Figure 2:**
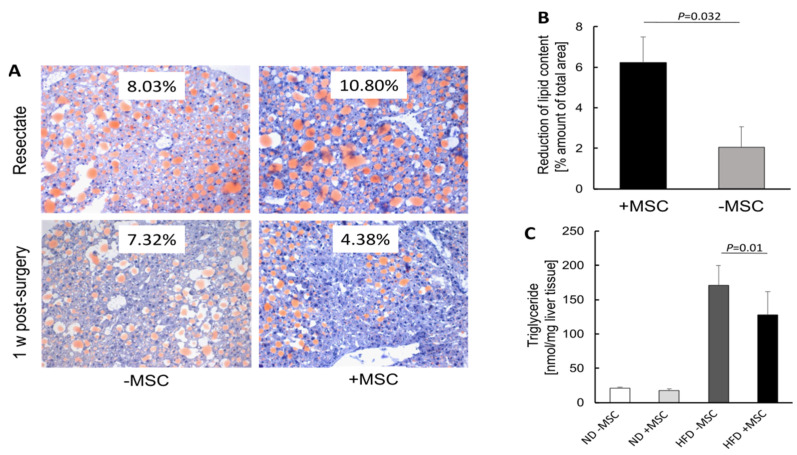
MSC treatment ameliorates lipid content in livers of high-fat-diet-fed mice; (**A**) lipid droplets were detected histochemically by staining with Sudan III in liver slices from the resectates before PBS (−MSC) or MSC (+MSC) application (cf. Materials and Methods) and 1 week post-surgery; pictures are representative for 5 and 6 different animals in the MSC group and in the group without MSC treatment, respectively; the percentage amount of lipid is shown in the insets (orig. magnification: 20×); (**B**) quantitative image analysis of pictures shown representatively in (**A**); values are means ± SEM and were calculated as the difference between lipid content at the time of surgery (resectate) and 1 week after application of either PBS (−MSC) or MSC (+MSC). This calculation was introduced to account for the different starting points in different animals featuring various degrees of NASH; (**C**) liver triglycerides were determined biochemically using the Triglyceride Assay Kit (Abcam); values are means ± SD from 3 (ND − MSC), 4 (ND +MSC), 9 (HFD − MSC) and 9 (HFD + MSC) animals per group; in (**B**,**C**), the two-sided Student´s *t*-test was applied for calculation of significance.

To substantiate this observational conclusion, we determined the hepatic lipid content by quantitative image analysis of Sudan III-stained cryosections before and 1 week after transplantation of MSC. Livers before transplantation of MSC feature similar amounts of Sudan III-stained lipid droplets. One week after MSC treatment, the decrease of fat content was three times higher as compared to livers without MSC treatment (Figure 2A,B). According to the biochemical analysis, triglycerides were 25% lower in livers of animals treated with MSC compared to untreated animals (Figure 2C). The decrease in lipid content by MSC treatment was also corroborated by fluorescence immunohistochemical detection of perilipin 1, a component of the lipid/protein coating of intracellular lipid droplets [36,37]. Perilipin 1 was clearly less abundant in NASH livers treated with MSC indicating lower lipid content compared to livers without treatment (Supplementary Figure S1).

### 3.2. MSC-Treatment Augments Expression of Lipid Utilization Genes

**Figure 3:**
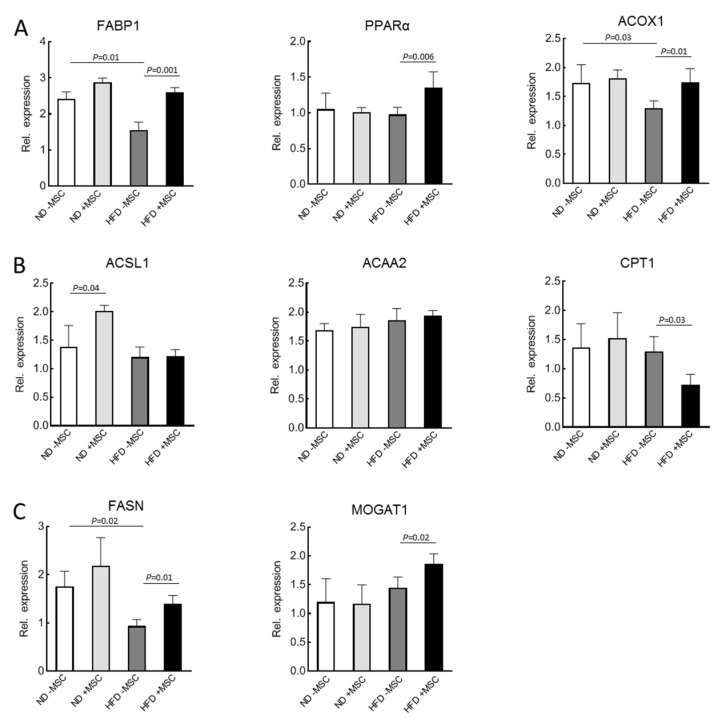
The mRNA expression of genes related to: (**A**) lipid utilization: FABP1, PPARα, ACOX1; (**B**) β-oxidation: ACSL1, ACAA2 and CPT1; and (**C**) triglyceride synthesis: FASN and MOGAT1 in livers of high-fat-diet-fed mice (HFD) treated with human MSC (+MSC); quantification of mRNA abundance by sqRT-PCR followed by densitometry; expression was normalized to the abundance of β2-microglobulin used as a housekeeping gene; values are means ± SD from 3 (ND − MSC), 4 (ND + MSC), 6 (HFD − MSC) and 8 (HFD + MSC) animals per group. Significant differences between the groups were validated by one-way ANOVA and the Newmann–Keuls test (SigmaStat; Jandel Scientific, San Rafael, CA, USA).

To gain mechanistic insight into the amelioration of lipid content by MSC treatment, the expression of mouse key lipid metabolism genes was analyzed by sqRT-PCR. In the liver, PPARα is the master regulator of lipid utilization [38]. Albeit not affected by feeding the high fat diet, its expression was upregulated by treatment with MSC. Consistently, expression of genes involved in lipid utilization, which are under the control of PPARα, such as fatty acid binding protein 1 (FABP1), fatty acid synthase (FASN) and acyl-CoA oxidase 1 (ACOX1), are upregulated by MSC treatment. Thus, downregulation by high fat diet feeding was corrected to almost normal as seen in animals without NASH (Figure 3A). Expression of genes involved in oxidation of fatty acids such as long-chain acyl-CoA synthetase (ACSL1) or acetyl-CoA acyltransferase (ACAA2) were neither downregulated in NASH livers, nor did MSC treatment affect expression. This indicates that utilization via β-oxidation by mouse mitochondria was probably not impacted by MSC treatment. Interestingly, carnitine palmitoyltransferase 1 (CPT1), the control enzyme of mitochondrial fatty acid import, was even downregulated by MSC treatment in the NASH livers (Figure 3B). In the context of MSC-mediated upregulation of FASN and monoacylglycerol acyltransferase 1 (MOGAT1) in the NASH livers (Figure 3C), it may be suggested that MSC treatment fuels fatty acids into triglyceride and VLDL synthesis and secretion, thus accounting for the attenuating of lipid content in the NASH livers with MSC treatment (cf. Figure 2). This, however, must be corroborated by the measurement of flux rates through the respective pathways of lipid utilization and storage. Expression of the respective human genes was not detected by using human-specific primer pairs for sqRT-PCR. Thus, amelioration of lipid load in the mouse livers by transplanted MSC did not involve lipid metabolism in the human cell transplants.

### 3.3. MSC Treatment Improves Tissue Homeostasis in NASH Livers

**Figure 4:**
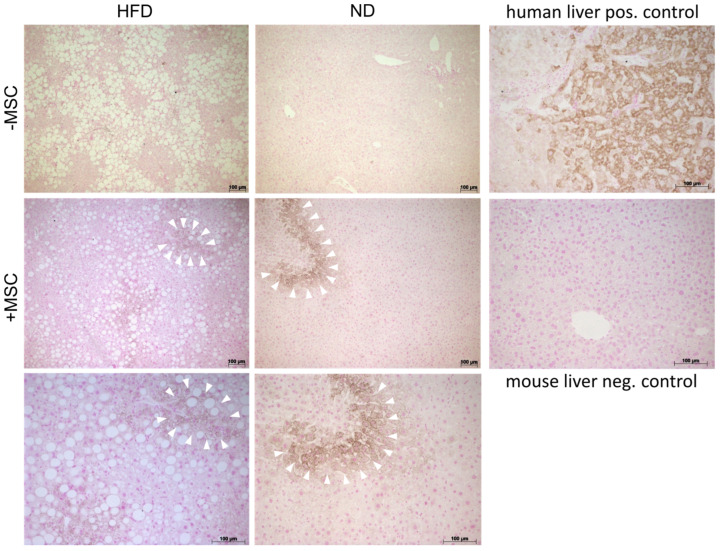
Human donor MSC in recipient livers of high-fat-diet-fed mice. Immunohistochemical detection of carbamoylphosphate synthetase 1 in human MSC transplants using the anti-human specific HepPar1 antibody reveals a patchy localization of the transplanted MSC (+MSC) both in livers without (ND) and with NASH (HFD). White arrowheads in the middle and bottom panels demarcate areas in the mouse parenchyma harboring transplanted human MSC. Bottom panels show higher magnifications (orig. mag. 20×) of the panels above (orig. mag. 10×). Pictures are representative for 3 different animals out of each group. Panels on the right show positive and negative control stainings in human and mouse liver tissue. Scale bar—100 µm.

First, we documented the presence of human MSC transplants in the mouse livers. By using the anti-human specific antibody HepPar1, which detects the urea cycle entry enzyme carbamoylphosphate synthetase 1 [39], human MSC were visible in the parenchyma of both control animals without NASH and animals fed the high fat diet featuring NASH. Visual estimation revealed bona fide similar amounts of MSC in control and NASH livers, but roughly less lipid storage in NASH livers treated with MSC (Figure 4), thus supporting biochemical data as shown in Figure 2C.

**Figure 5:**
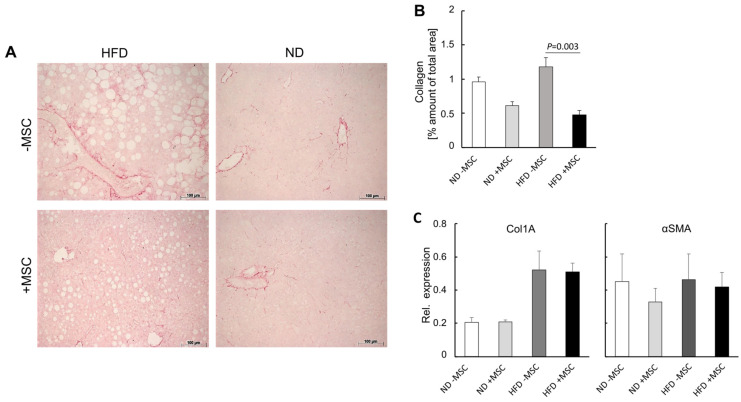
MSC treatment ameliorates fibrosis in livers of high-fat-diet-fed mice: (**A**) collagen was detected histochemically by staining with Sirius red in liver slices from animals either fed the high fat diet (HFD) or not (ND); where indicated, mice were treated with human MSC (+MSC) or left untreated (−MSC); pictures are representative for 3 different animals out of each group (orig. magnification: 20×; Scale bar—100 µm); (**B**) quantitative image analysis of pictures shown representatively in (**A**); values are means ± SD from 3 (ND − MSC), 4 (ND + MSC), 6 (HFD − MSC) and 8 (HFD + MSC) animals per group; from each animal, 10 tissue slices were analyzed using ImageJ as described; statistics were calculated with SPSS 23; after evaluation of standard distribution, the Kruskal-Wallis algorithm and post hoc Bonferroni were applied for calculation of significance; (**C**) abundance of collagen 1A1 (Col1A) and αSMA mRNA as assessed by sqRT-PCR; expression was normalized to the expression of Hprt1 (hypoxanthine phosphoribosyltransferase 1) used as a housekeeping gene; values are means ± SEM from 3 (ND − MSC), 4 (ND + MSC), 5 (HFD − MSC) and 6 (HFD + MSC) animals per group.

Second, we quantified collagen deposition, which increases during NASH progression [40]. As evidenced by quantitative image analysis of Sirius red-stained tissue slices, collagen in the liver was elevated by about 25%, though not significantly, in animals fed the high fat diet compared with control animals. Treatment with MSC decreased collagen by about one half both in animals fed the control diet and in high-fat-diet-fed animals (Figure 5A,B). The increase in collagen was substantiated by the increase in collagen 1A mRNA after feeding the high fat diet. However, this increase was not attenuated by MSC treatment (Figure 5C, left); αSMA expression, which indicates activation of hepatic stellate cells and collagen production, was also not affected by MSC treatment (Figure 5C, right). This corroborates previous data shown in rats and mice with CCl_4_-induced liver cirrhosis. In these studies, the decrease in collagen as quantified by image analysis was rather due to tissue re-distribution of fibrous to fibrillar collagen than to a decrease of its synthesis and amount [19,41].

**Figure 6:**
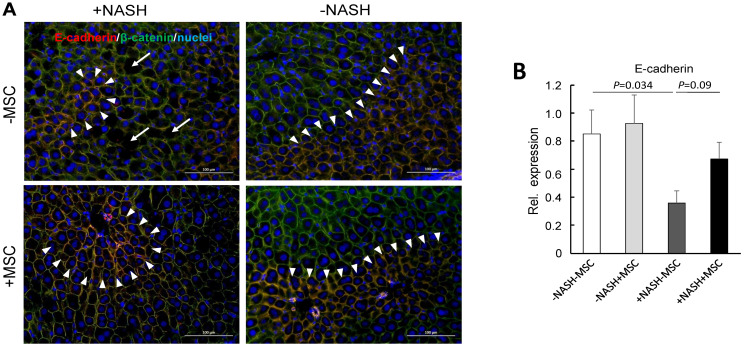
MSC treatment restores tissue homeostasis in livers of high-fat-diet-fed mice; (**A**) E-cadherin (red) and β-catenin (green) were detected by fluorescent immunohistochemistry in liver slices from animals either fed the high fat diet (HFD) or not (ND); where indicated, mice were treated with human MSC (+MSC) or left untreated (−MSC); pictures are representative for 3 different animals out of each group (orig. magnification: 20×; Scale bar—100 µm); white arrowheads demarcate zonal co-expression of E-cadherin and β-catenin (yellow = red/green overlay) in periportal hepatocytes, while β-catenin is expressed pan-parenchymally; blue, nuclear stain with DAPI. White arrows indicate “holes” of lipid deposition in the NASH livers; (**B**) quantification of E-cadherin mRNA abundance by sqRT-PCR; expression was normalized to the expression of β2-microglobulin used as a housekeeping gene; values are means ± SEM from 3 (ND − MSC), 4 (ND + MSC), 6 (HFD − MSC) and 6 (HFD + MSC) animals per group; one-way ANOVA and LSD post hoc test to calculate significance between groups.

In the healthy mouse liver, the adherens junction proteins E-cadherin and β-catenin are expressed in periportal hepatocytes and over the entire parenchyma, respectively, [42]. This pattern was also observed here in mouse livers without NASH irrespective of treatment with or without MSC (Figure 6A, right panels). In the NASH livers, E-cadherin expression was decreased and restricted to single layers of periportal hepatocytes. Broad extension of expression similar to controls was preserved by treatment with MSC (Figure 6A, upper left vs. lower left panel). Expression of E-cadherin was significantly downregulated on the transcriptional level as verified by sqRT-PCR. Expression was restored in part by treatment with MSC (Figure 6B). These findings confirm previous data in a mouse model of MCD-diet-induced NASH showing disruption of E-cadherin and β-catenin co-expression in periportal hepatocytes indicative of loss of the epithelial integrity and hepatocyte polarity [30] and hepatocyte functions [43].

This indicates epithelial–mesenchymal transition in the NASH livers, which is also underlined by the expression of N-cadherin and ZO-1, parenchymal adherens and tight junction proteins, respectively. In the control livers without NASH, ZO-1 and N-cadherin were expressed pan-parenchymally. In the NASH livers, both proteins were significantly reduced in the untreated NASH livers, leaving only small areas of expression. Expression was largely preserved in livers of animals treated with MSC (Supplementary Figure S2).

Taken together, these data show that the high fat diet causes epithelial perturbation associated with an increase of collagen and fat deposition. While MSC treatment clearly ameliorates the lipid load, fibrotic changes were hardly affected by the treatment with MSC. However, MSC presumably preserved epithelial integrity and hence proper hepatocyte polarity and function.

### 3.4. MSC Ameliorate NASH-Associated Inflammation

**Figure 7:**
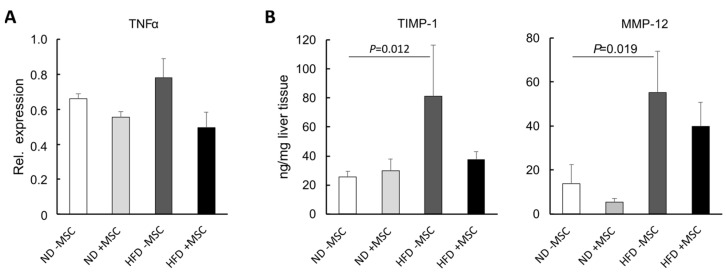
MSC treatment decreases molecular markers of inflammation in livers of high-fat-diet-fed mice:(**A**) quantification of TNFα mRNA abundance by sqRT-PCR; expression was normalized to the expression of hypoxanthine phosphoribosyltransferase (Hprt1) used as a housekeeping gene; values are means ± SEM from 3 (ND − MSC), 4 (ND + MSC), 5 (HFD − MSC) and 6 (HFD + MSC) animals per group; (**B**) TIMP-1 and MMP-12 were quantified by Multiplex analysis; values are means ± SEM from 4, 4 (ND − MSC), 4, 3 (ND + MSC), 5, 6 (HFD − MSC) and 7, 7 (HFD + MSC) animals per group for the determination of TIMP-1 and MMP-12, respectively; values are means ± SEM; statistics: LSD post hoc test after Johnson transformation.

On the tissue level, hepatic inflammation was still obvious in livers of high-fat-diet-fed mice even after MSC treatment (Figure 1B). However, on the molecular level, the increase in TNFα mRNA, a major mediator of hepatic inflammation in NASH, was reduced to normal by the treatment with MSC (Figure 7A). As determined by Multiplex analysis, TIMP-1 and MMP-12 were also elevated in the livers of mice fed the high fat diet compared to the controls, indicative for the onset of the acute-phase reaction, the hepatic defense response to injury and inflammation [44]. Again, this increase was attenuated by MSC treatment to levels not significantly different from the controls fed the normal diet (Figure 7B). Thus, MSC treatment attenuated the inflammatory response of the liver to the high fat diet feeding.

Thrombospondin-1 (THBS1) is elevated in fibrotic liver diseases [45] including NASH [46], and THBS1 from macrophages is assumed to foster NASH in the context of obesity [47]. Likewise, thrombocyte-derived THBS1 promotes acute tissue damage after extended liver surgery [48], altogether demonstrating that blood-borne cellular mechanisms drive the pathogenesis of NASH. In mice fed the high fat diet, THBS1 was elevated in the liver as verified by fluorescent immunohistochemistry (Supplementary Figure S3A). In line, thrombocytes, a major source of THBS1, and THBS1 mRNA transcripts were increased in NASH after feeding the high fat diet (Supplementary Figure S3A,B). Neither the increase in THBS1 mRNA and protein, nor that of thrombocytes were affected by the MSC treatment, indicating that MSC seem not to interfere directly with THBS1-driven mechanisms.

### 3.5. Human MSC Transplants Donate Mitochondria to Mouse Host Hepatocytes

In a previous study, we showed that human bone-marrow-derived MSC co-cultured with primary mouse hepatocytes delivered mitochondria to the hepatocytes, which likely supported lipid breakdown in the hepatocytes [30]. Here, we therefore assume that one potential mechanism of MSC-mediated amelioration of lipid load in livers of mice fed the high fat diet might involve the transfer of mitochondria derived from transplanted hBM-MSC to mouse hepatocytes. To support this assumption, we aimed at localization of human mitochondria in mouse hepatocytes by immunofluorescence histochemistry. We first tested the specificity of the antibody to detect human mitochondria. The anti-human-mitochondria antibody used here (for details cf. Table 1) identifies mitochondria in the human liver, but not in the mouse liver. To mark hepatocytes in the mouse livers, we used a specific anti-mouse-cyclophilin antibody, which does not yield any cross-reactivity with human liver (Supplementary Figure S4).

**Figure 8:**
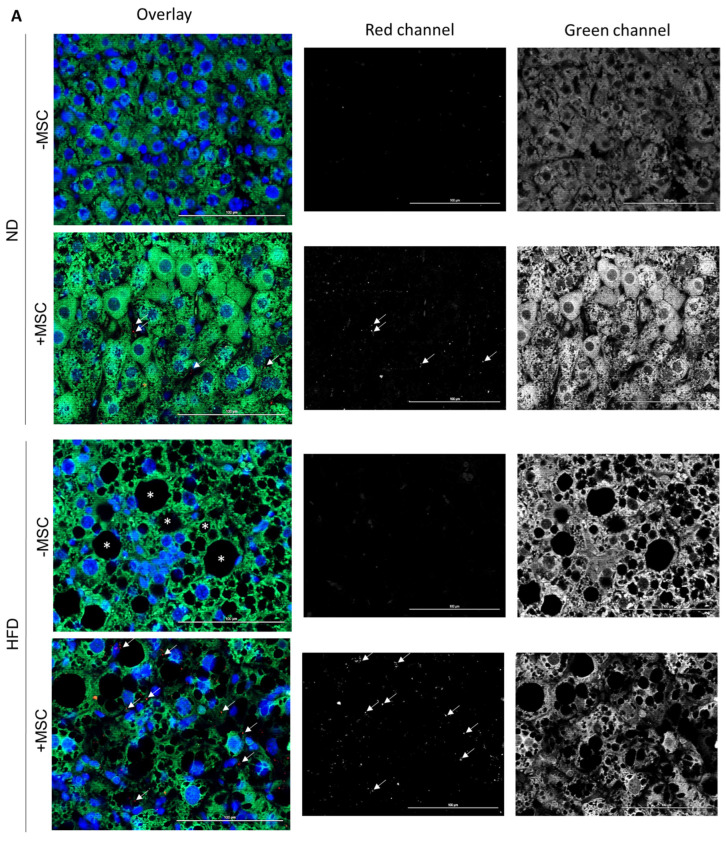

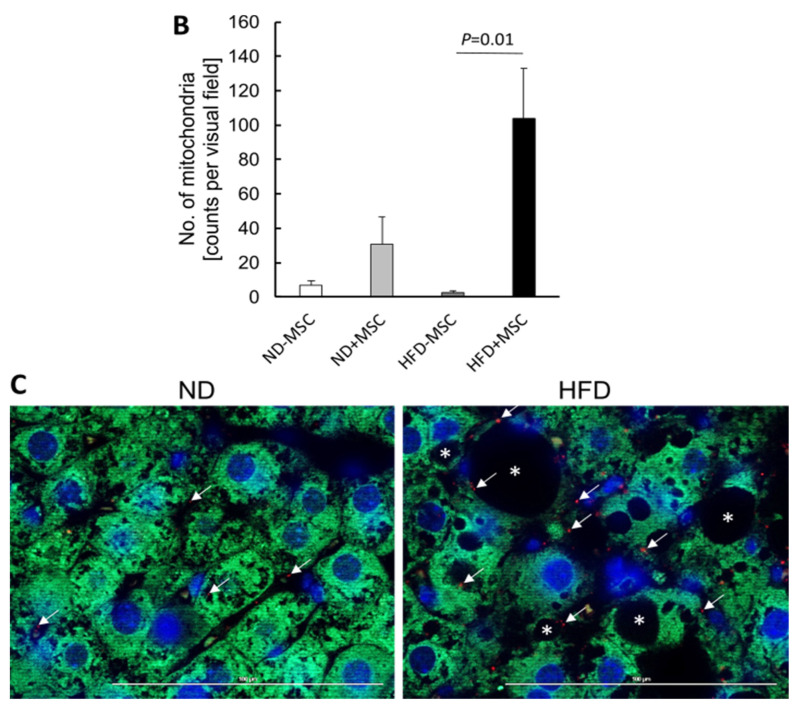
Human mitochondria derived from transplanted hBM-MSC in mouse hepatocytes. Mice fed the control (ND) or the high fat diet (HFD) were either treated (+MSC) or not (−MSC) with human bone-marrow-derived MSC. (**A**) Mouse cyclophilin (green in left column (overlay); white/grey in right column (green channel)) and human mitochondria (red in left column (overlay); white/grey in middle column (red channel)) were detected by immunofluorescence on liver tissue slices using species-specific antibodies. Images were taken with the Zeiss Axio Observer.Z1 microscope with ApoTome.2 at a 40× objective magnification. White arrows mark human mitochondria in livers receiving human MSC. Livers without MSC transplants did not display signals. Nuclei were stained with DAPI (blue). Asterisks label lipid droplets in livers of animals fed the high fat diet. Pictures are representative for livers from 2–3 animals in each group. (**B**) Quantitative image analysis of pictures shown representatively in (**A**). Values are mean counts ± SEM from 5 visual fields each on slices from 2 different animals in the (ND − MSC), (ND + MSC), (HFD − MSC) and from 7–8 visual fields each on slices from 3 different animals in the (HFD + MSC) group, respectively. Visual fields were analyzed using the ImageJ software as described under Materials and Methods. Statistics were calculated with SPSS 23. After evaluation of standard distribution, the Kruskal–Wallis algorithm and post hoc Bonferroni were applied for calculation of significance. (**C**) Representative images displaying the co-localization of mouse cyclophilin (green) and human mitochondria (red) in hepatocytes of mice receiving MSC transplants either fed the control diet (ND) or the high fat diet (HFD). Procedures were as described in (**A**) except that images were taken at the 63× objective magnification. Scale bars—100 µm.

Comparing livers from mice with and without MSC transplantation, human mitochondria were only detectable in livers with MSC transplants. Co-localization with mouse cyclophilin clearly indicates that human mitochondria reside in the mouse hepatocytes (Figure 8A). The number of mitochondria in a visual field (0.03763 mm^2^) was about 3.5 times higher in NASH livers (2763 per mm^2^) compared to normal livers without NASH (823 per mm^2^) (Figure 8B). Given the thickness of the sections used for mitochondria counting of 1 µm and the volume of a hepatocyte of 8 × 10^−6^ mm^3^ (volume of a cube with 20 µm edge length), MSC transplantation in the NASH livers yielded 587 human mitochondria per mouse hepatocyte. This is about one-fourth the total number of mitochondria in one hepatocyte (cf. https://www.biologie-schule.de/mitochondrium.php (accessed on 17 May 2022)). Assuming that donated mitochondria are solely engaged in lipid degradation via β-oxidation, they may thus account for the 25% decrease in triglycerides as shown in Figure 2C in NASH livers after MSC treatment. Higher magnification unravels that the human mitochondria often closely align with lipid droplets in the mouse hepatocyte cytoplasm (Figure 8C). This supports the assumption that transplanted MSC donate human mitochondria to mouse hepatocytes. Thus, in line with our previous data shown in a mouse model of MCD-diet-induced NASH, MSC-derived mitochondrial transfer may support lipid breakdown in the recipient mouse hepatocytes [30].

## 4. Discussion

Here, we show that treatment with human bone-marrow-derived mesenchymal stromal cells, before being differentiated into the hepatocytic phenotype, ameliorated lipid load and improved tissue homeostasis in the mouse liver suffering from NASH induced by feeding a high fat diet. Since donor MSC-derived mitochondria reside in recipient mouse hepatocytes, we suggest that donated mitochondria provide oxidative power for lipid breakdown, and thus support subsequent restoration of the tissue architecture.

### 4.1. Potential Mechanisms of MSC-Mediated Improvement of Hepatocyte Functions

Recent debate raises awareness that the pathogenesis of NASH is of multifactorial complexity driven by both a metabolic and a genetic pre-disposition [49,50,51,52]. Individual factors such as gender, age, social status, and environmental impacts, in addition to others, influence the manifestation of NASH [5,53]. Therefore, a multitude of distinct imprints of NASH and associating co-morbidities contributing to NASH still render understanding of the pathogenesis incomplete.

In the animal model used here, we may exclude anthropomorphic and genetic contributions to the manifestation of NASH in the mouse livers after feeding with the high fat diet in relation to the differences between treatment groups. Hence, excess lipid load, inflammation and tissue deterioration with loss of hepatocyte polarity and function marks solely the end point of mainly carbohydrate and lipid metabolism dysregulation. Adipose insulin resistance seems a major contributor to the rise in the blood of free fatty acids due to stimulated adipocyte lipolysis [54]. Free fatty acid utilization in the liver is under the control of the master transcription factor PPARα stimulating expression of genes involved in lipid utilization [38]. Feeding with a high fat diet obviously downregulates lipid utilization as indicated by the downregulation of PPARα. This may potentially be corrected by the MSC treatment through upregulation of PPARα and the normalization of expression of genes under the control of PPARα such as FABP1 engaged in intracellular fatty acid trafficking and like ACOX1 involved in peroxisomal β-oxidation. These were upregulated by MSC treatment, potentially indicating the shift of lipid storage to utilization. However, mitochondrial function seems neither affected in the NASH livers, nor by MSC treatment, as may be deduced from the unchanged expression of the mitochondrial proteins ACSL1 and ACAA2. This indicates that mouse mitochondria were not targets of MSC in this animal model. MSC even inhibit CPT1 expression, which is the mitochondrial import protein for fatty acids, while genes involved in de novo lipogenesis (FASN, MOGAT) were upregulated by the MSC (cf. Figure 3). Hence, MSC treatment increases genes involved in triglyceride synthesis while lipids simultaneously decrease. Taken together, this supports the hypothesis that MSC might improve NASH, possibly by fueling fatty acids into alternate usage pathways apart from mitochondrial β-oxidation and lipids into VLDL synthesis and secretion. This may indicate restoration of hepatocyte metabolic homeostasis by MSC, thus supporting tissue remodeling and repair. In line, diacylglycerol acyltransferase (DGAT) knockdown improved lipid depositions in db/db mice after induction of NASH by feeding a methionine/choline-deficient diet, albeit liver damage was aggravated. This suggested that triglyceride synthesis protected from liver damage [55]. Similarly, MSC improved lipid metabolism in the pig liver after ischemia reperfusion injury and hepatectomy by increasing, in addition to others, hepatic VLDL [56].

Excess fat in the liver stimulates lipid toxicity by the augmentation of oxidative stress emerging from enhanced peroxisomal and microsomal fatty acid oxidation. This drives hepatocyte dysfunction and devastation by protein and lipid peroxidation. Consecutive activation of inflammatory cells including stellate and Kupffer cells promotes inflammation and fibrosis [11,57]. The anti-inflammatory and anti-fibrotic effects of MSC are documented in various NASH models including high-fat-diet- [58,59] and MCD-diet-induced NASH [20,21]. In the NASH model used here, both fibrosis and inflammation were manifested only mildly when collagen distribution (cf. Figure 4) and TNFα expression (cf. Figure 6A) were considered as biochemical markers. Using elastin expression as a surrogate for extracellular matrix abundance, the ratio of TIMP-1:MMP-12 representatively demonstrated that the ultimate net abundance of fibrotic proteins depends on the balance and imbalance, respectively, of proteases and their inhibitors [60]. Thus, the decrease of the TIMP-1:MMP-12 ratio as shown here further suggests resolution of fibrosis by the MSC treatment.

As a consequence of hepatocyte damage by oxidative stress, the epithelial organization of the liver parenchyma becomes leaky as shown by the deterioration of zonal expression and the abundance of the adhesion proteins. This further aggravates hepatocyte functional impairment, which relies on the epithelial and polar organization of hepatocytes along the sinusoids [42,61]. MSC treatment preserves epithelial integrity. As shown recently, MSC did not directly interfere with epithelial adhesion contacts, but rather indirectly affected pathways promoting epithelial–mesenchymal transitions such as thrombospondin-1/TGFβ-1 [48]. THBS1 is not affected by MSC treatment in the present study, suggesting other pathways being involved and corroborating the assumption that improvement of epithelial integrity and hepatocyte functions is not the cause, but rather the consequence of MSC action.

### 4.2. Mitochondria Transfer from Human MSC to Mouse Hepatocytes

Probably, the MSC do not exert their pleiotropic effects on fibrosis and inflammation as well as on lipid reduction and restoration of hepatocyte epithelial organization via different modes of actions. It is rather conceivable that the overall improvement of tissue homeostasis follows the normalization of lipid metabolism and consecutive metabolic events such as oxidative stress. Mitochondrial dysfunction plays a central role in the pathogenesis of NASH as a result of fatty acid overload due to unrestricted lipolysis in the insulin-resistant adipose tissue. As a consequence, energy substrates fuel into storage rather than utilization [27,62]. In our study, the expression of genes involved in lipid storage and utilization may suggest that oxidative degradation of fatty acids in the mitochondria might not principally be impaired. As long as simple steatosis prevails, healthy mitochondria work at their oxidative limit to establish new homeostasis under high free fatty acid conditions. Mitochondrial dysfunction might emerge with the onset of inflammatory processes and oxidative stress [63,64]. The functional link between lipid metabolism and mitochondria is documented by the close vicinity of lipid droplets and mitochondria in hepatocytes. Contact proteins including perilipins are not only involved in mediating the membrane contact sites between lipid droplets and mitochondria, but they also participate in the regulation of lipid metabolism [37,65]. Mitochondria promote both lipid droplet size expansion and reduction by providing substrates for triglyceride synthesis and lipid degradation by triglyceride hydrolysis and oxidation of fatty acids, respectively, [66]. Therefore, the increase in perilipin-1 as shown here in the NASH livers indicates communication between lipid droplets and mitochondria likely in relation to lipid storage. The decrease in perilipin-1 in the context of human mitochondria in proximity of lipid droplets in mouse hepatocytes hence suggests that the donation of human MSC-derived mitochondria eventually mediates lipid breakdown by oxidative phosphorylation of triglyceride-derived fatty acids. This corroborates previous findings in primary mouse hepatocytes demonstrating transport of human MSC-derived mitochondria via tunneling nanotubes into mouse hepatocytes, which provided oxidative capacity for lipid breakdown. In this study, NASH was established by feeding mice a methionine/choline-deficient (MCD) diet. Inhibition of VLDL synthesis by the impairment of ApoB synthesis characterizes this NASH model. Albeit VLDL synthesis did not improve under continuation of MCD diet feeding, MSC treatment still ameliorated NASH [30].

## 5. Conclusions

Here we show that MSC treatment of NASH significantly ameliorates hepatic lipid load in mice. Feeding the HFD diet robustly induced NASH featuring hepatocyte steatosis and ballooning as well as tissue inflammation and fibrosis. All signs of NASH were reduced to normal, thus corroborating previous observations in a mouse model of MCD-diet-induced NASH. Since MSC improved NASH in both the MCD and the high fat diet model, featuring two different pathomechanisms, we suggest that delivery of human MSC-derived mitochondria to mouse hepatocytes is a feasible way to provide lipid utilization capacity by enhancing mitochondrial oxidative power independent of the different pathogenic events. However, the causal link between mitochondria delivery and enhancement of oxidative lipid breakdown still needs to be established. Attempts to manipulate mitochondrial activity by the use of inhibitors of mitochondria function failed in our hands because of the hepatotoxicity of the drugs commonly in use for this purpose. Nevertheless, mitochondria transfer to target cells has been widely observed as a novel mechanism of MSC action in addition to their known mode of action by tissue replacement and tropic paracrine actions [67,68,69]. Mitochondria transplantation has shown a broad therapeutic efficacy in animal studies, revealing, e.g., improvement of drug-induced kidney injury in rats [70], functional enhancement of the ischemic myocard in rabbits [71] and decrease of liver injury after ischemia/reperfusion in rats [72], just to mention a few. The widespread application of mitochondria transplantation underscores its versatility leading to clinical trials, e.g., showing in pediatric patients improvement of myocardial impairment by autologous mitochondria transplantation [73].

From our results, we therefore conclude that mitochondrial transfer from human MSC transplants to host mouse hepatocytes is the primary effect decreasing hepatic lipid load by providing oxidative capacity. Secondary, self-restoration by amelioration of NASH-associated fibrosis and inflammation eventually improve tissue and metabolic homeostasis.

## Figures and Tables

**Table 1:** List of antibodies used for immunohistochemistry.

Antigen	Cat. No., Supplier	Dilution
HepPar1 [OCH1E5]	MAB7927, Abnova, Taipeh, Taiwan	1:100
Perilipin 1	ab3526, Abcam, Cambridge, England	1:600
E-cadherin	610182, BD Biosciences, Heidelberg, Germany	1:200
β-catenin	610154, BD Biosciences, Heidelberg, Germany	1:200
THBS1	ab1823, Abcam, Cambridge, England	1:100
CD42b	ab183345, Abcam, Cambridge, England	1:200
N-cadherin	610921, BD Biosciences, Heidelberg, Germany	1:200
ZO-1	44-2200, InVitrogen, Carlsbad, CA, USA	1:200
Anti-human mitochondria	MAB1273, Millipore, Darmstadt, Germany	1:200
Cyclophilin	2175, Cell Signaling, Cambridge, England	1:100
HRP goat anti-mouse Ig	554002, BD Biosciences, Heidelberg, Germany	1:200
Goat anti-mouse Cy3	115-165-003, Dianova, Hamburg, Germany	1:200
Goat anti-rabbit AlexaFluor 488	A11008, Life Technologies, Ober-Olm, Germany	1:300
Goat anti-mouse AlexaFluor 488	115-545-003, Dianova, Hamburg, Germany	1:200

**Table 2:** List of human (h) and mouse (m) primer pairs used for sqRT-PCR.

Target Transcript	F (5′→3′)	R (5′→3′)	bp	h/m
ACAA2	GGGCACTGAAGAAAGCAGGA	CGTGAACCAGGTGTGCAGTA	198	h
	ACATAACTTCACGCCCCTGG	GCAAAAGCTTCGTTCACGTCT	162	m
ACOX1	GCCGCCGAGAGATCGAGAAC	CGCCCTCGGTGCACAAAA	193	h
	CGTCGAGAAATCGAGAACTTG	GGTTCCACAAAATTGACCATATGTA	200	m
ACSL1	GTGGAACTACAGGCAACCCC	ATCATCTGGGCAAGGATTGAC	113	h
	AAGTGGAACTACAGGCAACCC	AGCTCCATGACACAGCATTACA	190	m
CPT1	AGTTCTCTTGCCCTGAGACG	GGGGCTTTGGACCGGTTG	139	h
	CATTCCGCCGCCGCC	ACCAGTGATGATGCCATTCTTG	200	m
FABP1	GCTGGGTCCAAAGTGATCCA	TATGTCGCCGTTGAGTTCGG	165	h
	GGGAAAAAGTCAAGGCAGTCG	GACAATGTCGCCCAATGTCA	131	m
FASN	TATGAAGCCATCGTGGACGG	GAAGAAGGAGAGCCGGTTGG	183	h
	TGGATTACCCAAGCGGTCTG	AGTGTTCGTTCCTCGGAGTG	181	m
MOGAT1	GGTAGAGTTTGCACCGCTCA	CTGGGGTATGCCAGTCAAAGT	191	h
	GGCTCACCCAGGAACATTCA	GTGGCAAGGCTACTCCCATT	200	m
PPAR-α	GCCTCCTTCGGCGTTCG	GAGCTCCAAGCTACTGTGGT	148	h
	GGGAACTTAGAGGAGAGCCAAG	CAGTGGGGAGAGAGGACAGA	183	m
β2-microglobulin	TGTCTTTCAGCAAGGACTGGT	TTCAAACCTCCATGATGCTGC	161	h
	TCTACTGGGATCGAGACATGTGA	ATTGCTATTTCTTTCTGCGTGCAT	124	m

Abbreviations: ACAA2—Acetyl Coenzyme A Acyltransferase 2; ACOX1—Acyl-CoA Oxidase 1; ACSL1—Acyl-CoA Synthetase Long Chain Family Member 1; CPT1—Carnitine Palmitoyltransferase I; FABP1—Fatty Acid-Binding Protein 1; FASN—Fatty Acid Synthase; MOGAT1—Monoacylglycerol O-Acyltransferase 1; PPAR-α—Peroxisome Proliferator-Activated Receptor-Alpha; F—forward; R—reverse; h—human, m—mouse.

## Data Availability

Data is contained within the article. Primary source data are available on request from the corresponding author.

## Supplementary Materials

The following supporting information can be downloaded at: https://www.mdpi.com/article/10.3390/cells11111829/s1, Figure S1: MSC attenuate lipid droplet-associated expression of perilipin 1 in livers of high fat diet-treated mice; Figure S2: MSC treatment preserves cell-cell contacts in livers of high fat diet-fed mice; Figure S3: MSC treatment does not involve THBS1-mediated mechanisms in livers of high fat diet-fed mice; Figure S4: Specificity of the immunofluorescent detection of human MSC-derived mitochondria in mouse hepatocytes.

## References

[1] Younossi Z.M., Koenig A.B., Abdelatif D., Fazel Y., Henry L., Wymer M. (2016). Global epidemiology of nonalcoholic fatty liver disease-meta-analytic assessment of prevalence, incidence, and outcomes. Hepatology.

[2] Dietrich P., Hellerbrand C. (2014). Non-alcoholic fatty liver disease, obesity and the metabolic syndrome. Best Pract. Research. Clin. Gastroenterol..

[3] Tacke F., Weiskirchen R. (2021). Non-alcoholic fatty liver disease (nafld)/non-alcoholic steatohepatitis (nash)-related liver fibrosis: Mechanisms, treatment and prevention. Ann. Transl. Med..

[4] Hashimoto E., Yatsuji S., Tobari M., Taniai M., Torii N., Tokushige K., Shiratori K. (2009). Hepatocellular carcinoma in patients with nonalcoholic steatohepatitis. J. Gastroenterol..

[5] Younossi Z.M., Henry L. (2021). Epidemiology of non-alcoholic fatty liver disease and hepatocellular carcinoma. JHEP Rep. Innov. Hepatol..

[6] Roeb E. (2021). Non-alcoholic fatty liver diseases: Current challenges and future directions. Ann. Transl. Med..

[7] Tannapfel A., Denk H., Dienes H.P., Langner C., Schirmacher P., Trauner M., Flott-Rahmel B. (2011). Histopathological diagnosis of non-alcoholic and alcoholic fatty liver disease. Virchows Arch. Int. J. Pathol..

[8] Day C.P., Saksena S. (2002). Non-alcoholic steatohepatitis: Definitions and pathogenesis. J. Gastroenterol. Hepatol..

[9] Pascale A., Pais R., Ratziu V. (2010). An overview of nonalcoholic steatohepatitis: Past, present and future directions. J. Gastrointest. Liver Dis. JGLD.

[10] Bessone F., Razori M.V., Roma M.G. (2019). Molecular pathways of nonalcoholic fatty liver disease development and progression. Cell. Mol. Life Sci. CMLS.

[11] Kim J.Y., He F., Karin M. (2021). From liver fat to cancer: Perils of the western diet. Cancers.

[12] Loomba R., Friedman S.L., Shulman G.I. (2021). Mechanisms and disease consequences of nonalcoholic fatty liver disease. Cell.

[13] Pittenger M.F., Discher D.E., Peault B.M., Phinney D.G., Hare J.M., Caplan A.I. (2019). Mesenchymal stem cell perspective: Cell biology to clinical progress. NPJ Regen. Med..

[14] Le Blanc K., Davies L.C. (2015). Mesenchymal stromal cells and the innate immune response. Immunol. Lett..

[15] Newman R.E., Yoo D., LeRoux M.A., Danilkovitch-Miagkova A. (2009). Treatment of inflammatory diseases with mesenchymal stem cells. Inflamm. Allergy Drug Targets.

[16] Salomone F., Barbagallo I., Puzzo L., Piazza C., Li Volti G. (2013). Efficacy of adipose tissue-mesenchymal stem cell transplantation in rats with acetaminophen liver injury. Stem Cell Res..

[17] Stock P., Bruckner S., Winkler S., Dollinger M.M., Christ B. (2014). Human bone marrow mesenchymal stem cell-derived hepatocytes improve the mouse liver after acute acetaminophen intoxication by preventing progress of injury. Int. J. Mol. Sci..

[18] Tanimoto H., Terai S., Taro T., Murata Y., Fujisawa K., Yamamoto N., Sakaida I. (2013). Improvement of liver fibrosis by infusion of cultured cells derived from human bone marrow. Cell Tissue Res..

[19] Bruckner S., Zipprich A., Hempel M., Thonig A., Schwill F., Roderfeld M., Roeb E., Christ B. (2017). Improvement of portal venous pressure in cirrhotic rat livers by systemic treatment with adipose tissue-derived mesenchymal stromal cells. Cytotherapy.

[20] Wang H., Wang D., Yang L., Wang Y., Jia J., Na D., Chen H., Luo Y., Liu C. (2017). Compact bone-derived mesenchymal stem cells attenuate nonalcoholic steatohepatitis in a mouse model by modulation of cd4 cells differentiation. Int. Immunopharmacol..

[21] Winkler S., Borkham-Kamphorst E., Stock P., Bruckner S., Dollinger M., Weiskirchen R., Christ B. (2014). Human mesenchymal stem cells towards non-alcoholic steatohepatitis in an immunodeficient mouse model. Exp. Cell Res..

[22] Ezquer M., Ezquer F., Ricca M., Allers C., Conget P. (2011). Intravenous administration of multipotent stromal cells prevents the onset of non-alcoholic steatohepatitis in obese mice with metabolic syndrome. J. Hepatol..

[23] Lee C.W., Hsiao W.T., Lee O.K. (2017). Mesenchymal stromal cell-based therapies reduce obesity and metabolic syndromes induced by a high-fat diet. Transl. Res. J. Lab. Clin. Med..

[24] Domingues C.C., Kundu N., Kropotova Y., Ahmadi N., Sen S. (2019). Antioxidant-upregulated mesenchymal stem cells reduce inflammation and improve fatty liver disease in diet-induced obesity. Stem Cell Res. Ther..

[25] Li L., Zeng X., Liu Z., Chen X., Li L., Luo R., Liu X., Zhang J., Liu J., Lu Y. (2021). Mesenchymal stromal cells protect hepatocytes from lipotoxicity through alleviation of endoplasmic reticulum stress by restoring serca activity. J. Cell. Mol. Med..

[26] Yang M., Cui Y., Song J., Cui C., Wang L., Liang K., Wang C., Sha S., He Q., Hu H. (2021). Mesenchymal stem cell-conditioned medium improved mitochondrial function and alleviated inflammation and apoptosis in non-alcoholic fatty liver disease by regulating sirt1. Biochem. Biophys. Res. Commun..

[27] Nassir F., Ibdah J.A. (2014). Role of mitochondria in nonalcoholic fatty liver disease. Int. J. Mol. Sci..

[28] Stankova P., Kucera O., Peterova E., Elkalaf M., Rychtrmoc D., Melek J., Podhola M., Zubanova V., Cervinkova Z. (2021). Western diet decreases the liver mitochondrial oxidative flux of succinate: Insight from a murine nafld model. Int. J. Mol. Sci..

[29] Takeichi Y., Miyazawa T., Sakamoto S., Hanada Y., Wang L., Gotoh K., Uchida K., Katsuhara S., Sakamoto R., Ishihara T. (2021). Non-alcoholic fatty liver disease in mice with hepatocyte-specific deletion of mitochondrial fission factor. Diabetologia.

[30] Hsu M.J., Karkossa I., Schafer I., Christ M., Kuhne H., Schubert K., Rolle-Kampczyk U.E., Kalkhof S., Nickel S., Seibel P. (2020). Mitochondrial transfer by human mesenchymal stromal cells ameliorates hepatocyte lipid load in a mouse model of nash. Biomedicines.

[31] Stock P., Bruckner S., Ebensing S., Hempel M., Dollinger M.M., Christ B. (2010). The generation of hepatocytes from mesenchymal stem cells and engraftment into murine liver. Nat. Protoc..

[32] Aurich I., Mueller L.P., Aurich H., Luetzkendorf J., Tisljar K., Dollinger M.M., Schormann W., Walldorf J., Hengstler J.G., Fleig W.E. (2007). Functional integration of hepatocytes derived from human mesenchymal stem cells into mouse livers. Gut.

[33] Winkler S., Hempel M., Bruckner S., Tautenhahn H.M., Kaufmann R., Christ B. (2016). Identification of pathways in liver repair potentially targeted by secretory proteins from human mesenchymal stem cells. Int. J. Mol. Sci..

[34] Kleiner D.E., Brunt E.M., Van Natta M., Behling C., Contos M.J., Cummings O.W., Ferrell L.D., Liu Y.C., Torbenson M.S., Unalp-Arida A. (2005). Design and validation of a histological scoring system for nonalcoholic fatty liver disease. Hepatology.

[35] Bedossa P., Poitou C., Veyrie N., Bouillot J.L., Basdevant A., Paradis V., Tordjman J., Clement K. (2012). Histopathological algorithm and scoring system for evaluation of liver lesions in morbidly obese patients. Hepatology.

[36] Filali-Mouncef Y., Hunter C., Roccio F., Zagkou S., Dupont N., Primard C., Proikas-Cezanne T., Reggiori F. (2021). The menage a trois of autophagy, lipid droplets and liver disease. Autophagy.

[37] Irungbam K., Churin Y., Matono T., Weglage J., Ocker M., Glebe D., Hardt M., Koeppel A., Roderfeld M., Roeb E. (2020). Cannabinoid receptor 1 knockout alleviates hepatic steatosis by downregulating perilipin 2. Lab. Investig..

[38] Wang Y., Nakajima T., Gonzalez F.J., Tanaka N. (2020). Ppars as metabolic regulators in the liver: Lessons from liver-specific ppar-null mice. Int. J. Mol. Sci..

[39] Shah S.S., Chandan V.S. (2018). Hepatocyte antigen expression in barrett esophagus and associated neoplasia. Appl. Immunohistochem. Mol. Morphol. AIMM.

[40] Hagstrom H., Nasr P., Ekstedt M., Hammar U., Stal P., Hultcrantz R., Kechagias S. (2017). Fibrosis stage but not nash predicts mortality and time to development of severe liver disease in biopsy-proven nafld. J. Hepatol..

[41] Popov Y., Sverdlov D.Y., Sharma A.K., Bhaskar K.R., Li S., Freitag T.L., Lee J., Dieterich W., Melino G., Schuppan D. (2011). Tissue transglutaminase does not affect fibrotic matrix stability or regression of liver fibrosis in mice. Gastroenterology.

[42] Hempel M., Schmitz A., Winkler S., Kucukoglu O., Bruckner S., Niessen C., Christ B. (2015). Pathological implications of cadherin zonation in mouse liver. Cell. Mol. Life Sci. CMLS.

[43] Xie G., Diehl A.M. (2013). Evidence for and against epithelial-to-mesenchymal transition in the liver. Am. J. Physiol. Gastrointest. Liver Physiol..

[44] Roeb E., Graeve L., Mullberg J., Matern S., Rose-John S. (1994). Timp-1 protein expression is stimulated by il-1 beta and il-6 in primary rat hepatocytes. FEBS Lett..

[45] El-Youssef M., Mu Y., Huang L., Stellmach V., Crawford S.E. (1999). Increased expression of transforming growth factor-beta1 and thrombospondin-1 in congenital hepatic fibrosis: Possible role of the hepatic stellate cell. J. Pediatric Gastroenterol. Nutr..

[46] Min-DeBartolo J., Schlerman F., Akare S., Wang J., McMahon J., Zhan Y., Syed J., He W., Zhang B., Martinez R.V. (2019). Thrombospondin-i is a critical modulator in non-alcoholic steatohepatitis (nash). PLoS ONE.

[47] Gwag T., Reddy Mooli R.G., Li D., Lee S., Lee E.Y., Wang S. (2021). Macrophage-derived thrombospondin 1 promotes obesity-associated non-alcoholic fatty liver disease. JHEP Rep. Innov. Hepatol..

[48] Nickel S., Vlaic S., Christ M., Schubert K., Henschler R., Tautenhahn F., Burger C., Kuhne H., Erler S., Roth A. (2021). Mesenchymal stromal cells mitigate liver damage after extended resection in the pig by modulating thrombospondin-1/tgf-beta. NPJ Regen. Med..

[49] Luukkonen P.K., Qadri S., Ahlholm N., Porthan K., Mannisto V., Sammalkorpi H., Penttila A.K., Hakkarainen A., Lehtimaki T.E., Gaggini M. (2022). Distinct contributions of metabolic dysfunction and genetic risk factors in the pathogenesis of non-alcoholic fatty liver disease. J. Hepatol..

[50] Al-Sulaiti H., Diboun I., Agha M.V., Mohamed F.F.S., Atkin S., Domling A.S., Elrayess M.A., Mazloum N.A. (2019). Metabolic signature of obesity-associated insulin resistance and type 2 diabetes. J. Transl. Med..

[51] Sliz E., Sebert S., Wurtz P., Kangas A.J., Soininen P., Lehtimaki T., Kahonen M., Viikari J., Mannikko M., Ala-Korpela M. (2018). Nafld risk alleles in pnpla3, tm6sf2, gckr and lyplal1 show divergent metabolic effects. Hum. Mol. Genet..

[52] Speliotes E.K., George J. (2022). Metabolic and genetic contributions to nafld: Really distinct and homogeneous?. J. Hepatol..

[53] Arrese M., Arab J.P., Barrera F., Kaufmann B., Valenti L., Feldstein A.E. (2021). Insights into nonalcoholic fatty-liver disease heterogeneity. Semin. Liver Dis..

[54] Guilherme A., Virbasius J.V., Puri V., Czech M.P. (2008). Adipocyte dysfunctions linking obesity to insulin resistance and type 2 diabetes. Nat. Reviews. Mol. Cell Biol..

[55] Yamaguchi K., Yang L., McCall S., Huang J., Yu X.X., Pandey S.K., Bhanot S., Monia B.P., Li Y.X., Diehl A.M. (2007). Inhibiting triglyceride synthesis improves hepatic steatosis but exacerbates liver damage and fibrosis in obese mice with nonalcoholic steatohepatitis. Hepatology.

[56] Zhang Q., Liu X., Piao C., Jiao Z., Ma Y., Wang Y., Liu T., Xu J., Wang H. (2022). Effect of conditioned medium from adipose derived mesenchymal stem cells on endoplasmic reticulum stress and lipid metabolism after hepatic ischemia reperfusion injury and hepatectomy in swine. Life Sci..

[57] Schwabe R.F., Tabas I., Pajvani U.B. (2020). Mechanisms of fibrosis development in nonalcoholic steatohepatitis. Gastroenterology.

[58] El-Derany M.O., AbdelHamid S.G. (2021). Upregulation of mir-96-5p by bone marrow mesenchymal stem cells and their exosomes alleviate non-alcoholic steatohepatitis: Emphasis on caspase-2 signaling inhibition. Biochem. Pharmacol..

[59] Ohara M., Ohnishi S., Hosono H., Yamamoto K., Yuyama K., Nakamura H., Fu Q., Maehara O., Suda G., Sakamoto N. (2018). Extracellular vesicles from amnion-derived mesenchymal stem cells ameliorate hepatic inflammation and fibrosis in rats. Stem Cells Int..

[60] Pellicoro A., Aucott R.L., Ramachandran P., Robson A.J., Fallowfield J.A., Snowdon V.K., Hartland S.N., Vernon M., Duffield J.S., Benyon R.C. (2012). Elastin accumulation is regulated at the level of degradation by macrophage metalloelastase (mmp-12) during experimental liver fibrosis. Hepatology.

[61] Oh S.H., Swiderska-Syn M., Jewell M.L., Premont R.T., Diehl A.M. (2018). Liver regeneration requires yap1-tgfbeta-dependent epithelial-mesenchymal transition in hepatocytes. J. Hepatol..

[62] Di Ciaula A., Passarella S., Shanmugam H., Noviello M., Bonfrate L., Wang D.Q., Portincasa P. (2021). Nonalcoholic fatty liver disease (nafld). Mitochondria as players and targets of therapies?. Int. J. Mol. Sci..

[63] Sanyal A.J., Campbell-Sargent C., Mirshahi F., Rizzo W.B., Contos M.J., Sterling R.K., Luketic V.A., Shiffman M.L., Clore J.N. (2001). Nonalcoholic steatohepatitis: Association of insulin resistance and mitochondrial abnormalities. Gastroenterology.

[64] Pessayre D., Fromenty B. (2005). Nash: A mitochondrial disease. J. Hepatol..

[65] Ma X., Qian H., Chen A., Ni H.M., Ding W.X. (2021). Perspectives on mitochondria-er and mitochondria-lipid droplet contact in hepatocytes and hepatic lipid metabolism. Cells.

[66] Benador I.Y., Veliova M., Liesa M., Shirihai O.S. (2019). Mitochondria bound to lipid droplets: Where mitochondrial dynamics regulate lipid storage and utilization. Cell Metab..

[67] Hsu Y.C., Wu Y.T., Yu T.H., Wei Y.H. (2016). Mitochondria in mesenchymal stem cell biology and cell therapy: From cellular differentiation to mitochondrial transfer. Semin. Cell Dev. Biol..

[68] Velarde F., Ezquerra S., Delbruyere X., Caicedo A., Hidalgo Y., Khoury M. (2022). Mesenchymal stem cell-mediated transfer of mitochondria: Mechanisms and functional impact. Cell. Mol. Life Sci. CMLS.

[69] Gomzikova M.O., James V., Rizvanov A.A. (2021). Mitochondria donation by mesenchymal stem cells: Current understanding and mitochondria transplantation strategies. Front. Cell Dev. Biol..

[70] Kubat G.B., Ozler M., Ulger O., Ekinci O., Atalay O., Celik E., Safali M., Budak M.T. (2021). The effects of mesenchymal stem cell mitochondrial transplantation on doxorubicin-mediated nephrotoxicity in rats. J. Biochem. Mol. Toxicol..

[71] McCully J.D., Cowan D.B., Pacak C.A., Toumpoulis I.K., Dayalan H., Levitsky S. (2009). Injection of isolated mitochondria during early reperfusion for cardioprotection. Am. J. Physiology. Heart Circ. Physiol..

[72] Lin H.C., Liu S.Y., Lai H.S., Lai I.R. (2013). Isolated mitochondria infusion mitigates ischemia-reperfusion injury of the liver in rats. Shock.

[73] Emani S.M., Piekarski B.L., Harrild D., Del Nido P.J., McCully J.D. (2017). Autologous mitochondrial transplantation for dysfunction after ischemia-reperfusion injury. J. Thorac. Cardiovasc. Surg..

