# Supplementary material for: Human Mesenchymal Stromal Cells Resolve Lipid Load in High Fat Diet-Induced Non-Alcoholic Steatohepatitis in Mice by Mitochondria Donation
Source: Cells. 2022 Jun 2;11(11):1829. doi: 10.3390/cells11111829 (PMC9180625; doi:10.3390/cells11111829)
Supplement: Supplementary file 1 [file cells-11-01829-s001.zip › cells-1691524-supplementary.pdf]

Human mesenchymal stromal cells resolve lipid load in high fat diet-induced non-alcoholic steatohepatitis in mice by mitochondria donation

Sandra Nickel, Madlen Christ, Sandra Schmidt, Joanna Kosacka, Hagen Kühne, Martin Roderfeld, Thomas Longerich, Lysann Tietze, Ina Bosse, Mei-Ju Hsu, Peggy Stock, Elke Roeb, and Bruno Christ

Supplementary file 1

Figure S1. MSC attenuate lipid droplet-associated expression of perilipin 1 in livers of high fat diet-treated mice.

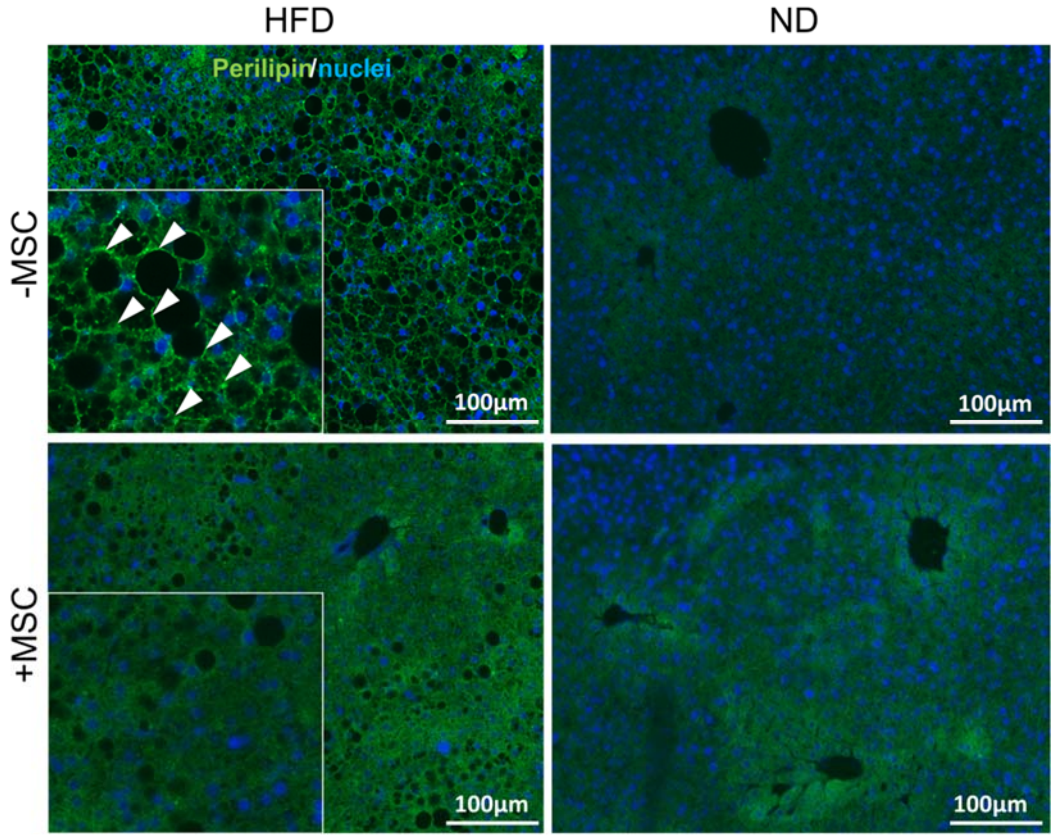

Immunohistochemical detection indicates increase of perilipin 1 expression in the periphery of lipid droplets in NASH livers (HFD; white arrowheads) as compared to livers without NASH (ND). MSC treatment (+MSC) reduces this increase. Left: inserted panels show digital magnifications of the images underlying (orig. mag. 20x). Pictures are representative for 3 different animals out of each group. Scale bar – 100 µm.

Figure S2. MSC treatment preserves cell-cell contacts in livers of high fat diet-fed mice.

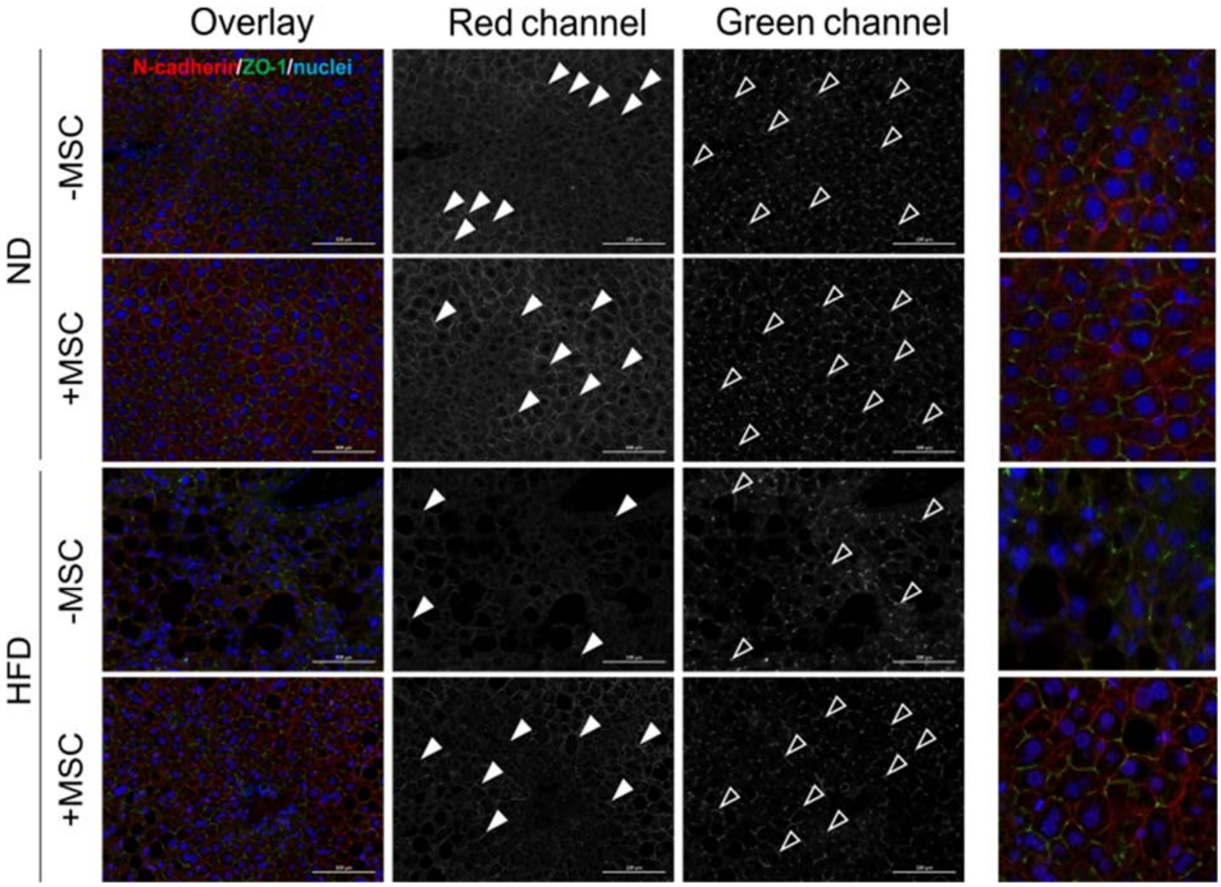

N-cadherin (red) and ZO-1 (green) were detected by fluorescent immunohistochemistry in liver slices from animals either fed the high fat diet (HFD) or not (ND). Where indicated, mice were treated with human MSC (+MSC), or left untreated (-MSC). Pictures are representative for 3 different animals out of each group (orig. magnification: 20x; Scale bar – 100 µm). N-cadherin (red channel, solid arrowheads) and ZO-1 (green channel, open arrowheads) are expressed pan-parenchymally featuring a slight preference of N-cadherin in pericentral hepatocytes. Blue, nuclear stain with DAPI. Pictures in the column at the right show digital cutout magnifications of the overlay pictures in the left column. Of note, N-cadherin expression (in red, third line of pictures) is nearly abrogated in NASH livers without MSC treatment.

Figure S3. MSC treatment does not involve THBS1-mediated mechanisms in livers of high fat diet-fed mice.

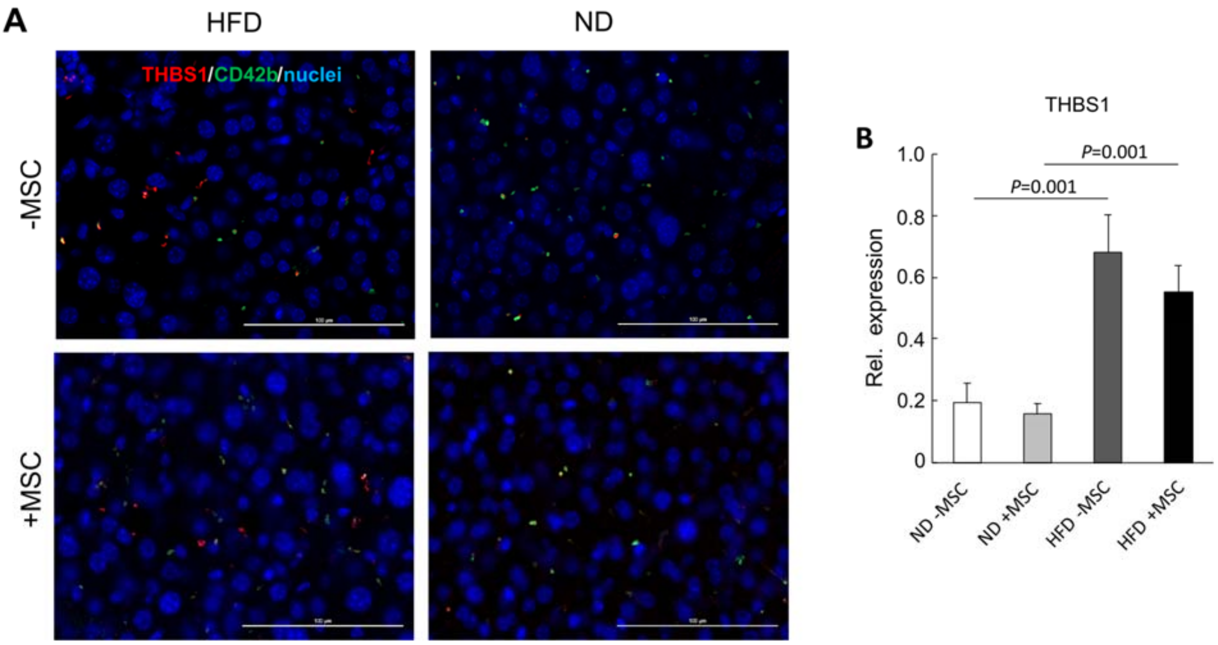

(A) THBS1 (red) and CD42b (green, thrombocyte marker) were detected by fluorescent immunohistochemistry in liver slices from animals either fed the high fat diet (HFD) or not (ND). Where indicated, mice were treated with human MSC (+MSC), or left untreated (-MSC). Pictures are representative for 3 different animals out of each group (orig. magnification: 40x; Scale bar – 100 µm). Note that THBS1 and CD42b largely do not overlap indicating that thrombocytes are not a major source of THBS1 in the liver. Blue, nuclear stain with DAPI. (B) Quantification of THBS1 mRNA abundance by sqRT-PCR. Expression was normalized to the expression of β2-microglobulin used as a housekeeping gene. Values are means±SEM from 3 (ND -MSC), 4 (ND +MSC), 7 (HFD -MSC), and 8 (HFD +MSC) animals per group, each run with RNA out of two different tissue pieces. Significant differences were calculated by one-way ANOVA and the LSD post-hoc test.

Figure S4. Specificity of the immunofluorescent detection of human MSC-derived mitochondria in mouse hepatocytes.

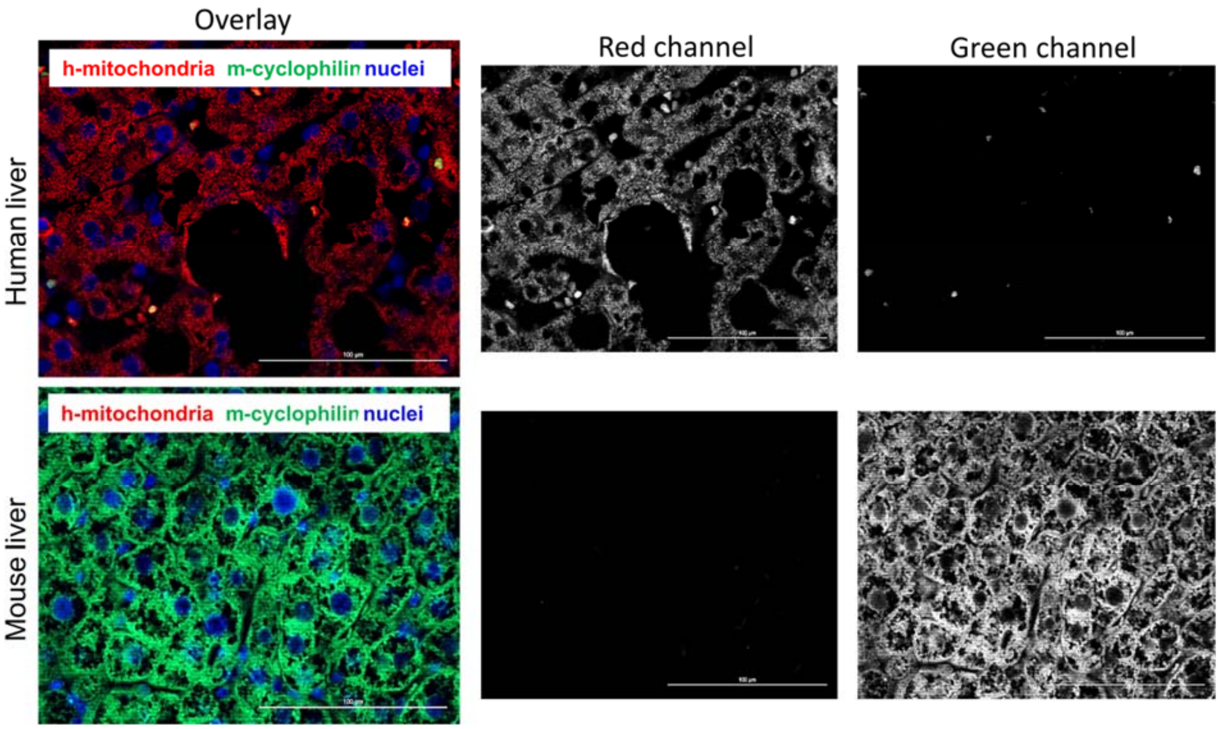

The anti-human-mitochondria antibody specifically detects human mitochondria in human liver slices (red in overlay/grey in red channel, upper panels) but not in mouse liver slices (left and middle lower panels). *Vice versa*, the anti-mouse-cyclophilin antibody identifies mouse cyclophilin in mouse liver slices (green in overlay/grey in green channel, bottom panels), but does not cross-react with human cyclophilin or any other human proteins (left and right upper panels). Original magnification – 40x; scale bars – 100 μm.
